# On the occasion of the world diabetes day 2013; diabetes education and prevention; a nephrology point of view

**DOI:** 10.12861/jrip.2013.11

**Published:** 2013-06-01

**Authors:** Hamid Nasri

**Affiliations:** ^1^Department of Nephrology, Division of Nephropathology, Isfahan University of Medical Sciences, Isfahan, Iran

**Keywords:** World diabetes day, Diabetic nephropathy, Chronic kidney disease

**Implication for health policy/practice/research/medical education:**

World diabetes day (WDD) is celebrated every year on November 14. Each year WDD is focused on a theme related to diabetes. Diabetes education and prevention is the WDD theme for the period 2009-2013.

One again, we reached to November 14, world diabetes day (WDD). WDD is celebrated every year on November 14(1). While diabetic nephropathy is a major cause of end-stage renal disease (ESRD), and the incidence of diabetes mellitus (DM) is growing rapidly. Many more people are at risk of type 2 diabetes and 470 million people globally will have pre-diabetes. As defined by the world health organization, individuals with pre-diabetes have fasting plasma glucose concentrations between 110 mg/dL and 126 mg/dL or as defined by the American Diabetes Association, between 101 mg/dL and 124 mg/dL(2-4).

Each year WDD is focused on a theme related to diabetes. WDD is an ideal opportunity to focus attention on diabetes, and to build alerts of the wide range of programs and services. Topics covered in the past have included diabetes and lifestyle, diabetes, human rights and the costs of diabetes(3-5).

Recent themes include:

2005: Diabetes and foot care
2006: Diabetes in the disadvantaged and the vulnerable
2007-2008: Diabetes in children and adolescents

Diabetes Education and prevention is the WDD theme for the period 2009-2013(1-6).

It seems that, WDD of 2013 is especial, while, in this period, the new pathological classification of diabetic nephropathy (DN) published by research committee of the renal pathology society in 2010. Pathologic classification of DN can strengthen the attentions to control and prevention of DN. DN is the most common cause of CKD and its number has been increasing. CKD is an international threat to health but the precise mechanism of this problem is not fully appreciated(7). DN affects around 40% of type 1 and type 2 diabetic patients. It increases the risk of death, mainly from heart problems, and is explained by increased urinary albumin excretion (UAE) in the absence of other renal diseases. DN is categorized into stages: microalbuminuria (UAE>20 μg/min and ≤199 μg/min) and macroalbuminuria (UAE ≥200 μg/min). Hyperglycemia, genetic predisposition and increased blood pressure levels are the main risk factors for the development of DN. In patients with type 2 diabetes, screening should be done at diagnosis and yearly thereafter(6-8). Patients with albuminuria should undergo an evaluation regarding the presence of comorbid associations, especially macrovascular disease and retinopathy. Achieving the best metabolic control (A1c <7%), treating high blood pressure (<130/80 mmHg or <125/75 mmHg if proteinuria >1.0 g/24 h and increased serum creatinine), using drugs with blockade effect on angiotensin II, and treating hyperlipidemia are effective plans for preventing the development of albuminuria, in delaying the progression to more advanced stages of diabetic kidney disease in patients with type 1 and type 2 diabetes. However, in recent years much attention have been directed toward better knowledge of morphologic lesions in DN and proposing a classification for DN to better control of this disease. In fact pathologic classifications exist for several kidney diseases such as lupus nephritis, focal segmental glomerulosclerosis and IgA nephropathy, yet there is no uniform classification for DN(7-9). Classification schemes lead to better communication between renal pathologists and clinicians, provide logistical structure for prognostic and interventional studies, and improve clinical management and efficiency. Also, the new pathological classification of DN can increase the diagnosis rate and attract more attention to tubular and interstitial injury in DN, providing to the early diagnosis and treatment of DN(7-12). However, it is necessary to combine morphological grading and biomarkers for DN to augment our understanding of this complex disease manifestation.

## Author’s contribution

HN is the single author of the manuscript.

## Conflict of interests

The author declared no competing interests.

## Ethical considerations

Ethical issues (including plagiarism, misconduct, data fabrication, falsification, double publication or submission, redundancy) have been completely observed by the authors.

## Funding/Support

None.

## References

[1] Schwandt P (2012). On the occasion of the world diabetes day: diabetes mellitus - a globally increasing health problem. Int J Prev Med.

[2] Platon I (2012). World Diabetes Day 2012--expanding the circle of influence. Diabetes Res Clin Pract.

[3] Jones  KL (2008). World Diabetes Day 2008: focusing on the children. Indian J Med Res.

[4] Silverstein JH, Kaufman FR (2008). World Diabetes Day--a global event for children. J Pediatr.

[5] Hirst MW, Felton A (2008). the UN Resolution on Diabetes and World Diabetes Day. Prim Care Diabetes.

[6] Yan L, Galvan A (2008). First World Diabetes Day calls for global action on disease. Nephrol News Issues.

[7] Fioretto P, Mauer M (2010). Diabetic nephropathy: diabetic nephropathy-challenges in pathologic classification. Nat Rev Nephrol.

[8] Kuzuya T, Nakagawa S, Satoh J, Kanazawa Y, Iwamoto Y, Kobayashi M (2002). Report of the Committee on the classification and diagnostic criteria of diabetes mellitus. Diabetes Res Clin Pract.

[9] Hollenberg NK (2001). Higher incidence of diabetic nephropathy in type 2 than in type 1 diabetes in early-onset diabetes in Japan. Curr Hypertens Rep.

[10] Oh SW, Kim S, Na KY, Chae DW, Kim S, Jin DC (2012). Clinical implications of pathologic diagnosis and classification for diabetic nephropathy. Diabetes Res Clin Pract.

[11] Valk EJ, Bruijn JA, BajemaIM BajemaIM (2011). Diabetic nephropathy in humans: pathologic diversity. Curr Opin Nephrol Hypertens.

[12] Tervaert TW, Mooyaart AL, Amann K, Cohen AH, Cook HT, Drachenberg CB (2010). Pathologic classification of diabetic nephropathy. J Am Soc Nephrol.

